# P-2196. Key Differences in Norovirus Genotypes during Cruise Ship Outbreaks, September 1, 2020 to March 31, 2025

**DOI:** 10.1093/ofid/ofaf695.2359

**Published:** 2026-01-11

**Authors:** Leigh Ellyn Preston, Stefanie White, Luis Rodriguez, Drew Kupper, Laura K Annetta, Erin Kincaid, Ronan King, James S Miller, Beth Osterink, Jessica Pharo, Shaun Stracener, Sydney Taylor

**Affiliations:** Vessel Sanitation Program, CDC, Atlanta, GA; Centers for Disease Control and Prevention, Atlanta, Georgia; Centers for Disease Control and Prevention, Atlanta, Georgia; Centers for Disease Control and Prevention, Atlanta, Georgia; Centers for Disease Control and Prevention, Atlanta, Georgia; Centers for Disease Control and Prevention, Atlanta, Georgia; Centers for Disease Control and Prevention, Atlanta, Georgia; Centers for Disease Control and Prevention, Atlanta, Georgia; Centers for Disease Control and Prevention, Atlanta, Georgia; Centers for Disease Control and Prevention, Atlanta, Georgia; Centers for Disease Control and Prevention, Atlanta, Georgia; Centers for Disease Control and Prevention, Atlanta, Georgia

## Abstract

**Background:**

CDC’s Vessel Sanitation Program (VSP) works with the cruise industry to monitor and investigate outbreaks of acute gastroenteritis (AGE) on cruise ships. Since 2019, 81% of cruise ship AGE outbreaks posted to VSP’s website were attributed to norovirus.^1,2^ Previously, most norovirus outbreaks in the United States (US) and cruise travel were attributed to the GII.4 genotype, recently, GII.17 has become the predominate genotype.^3^ Due to its new emergence in the US, characteristics of illness with GII.17 are not well described in adult populations. We compare case demographics and disease severity between GII.4 and GII.17 from cruise ship norovirus outbreaks.

**Figure:**
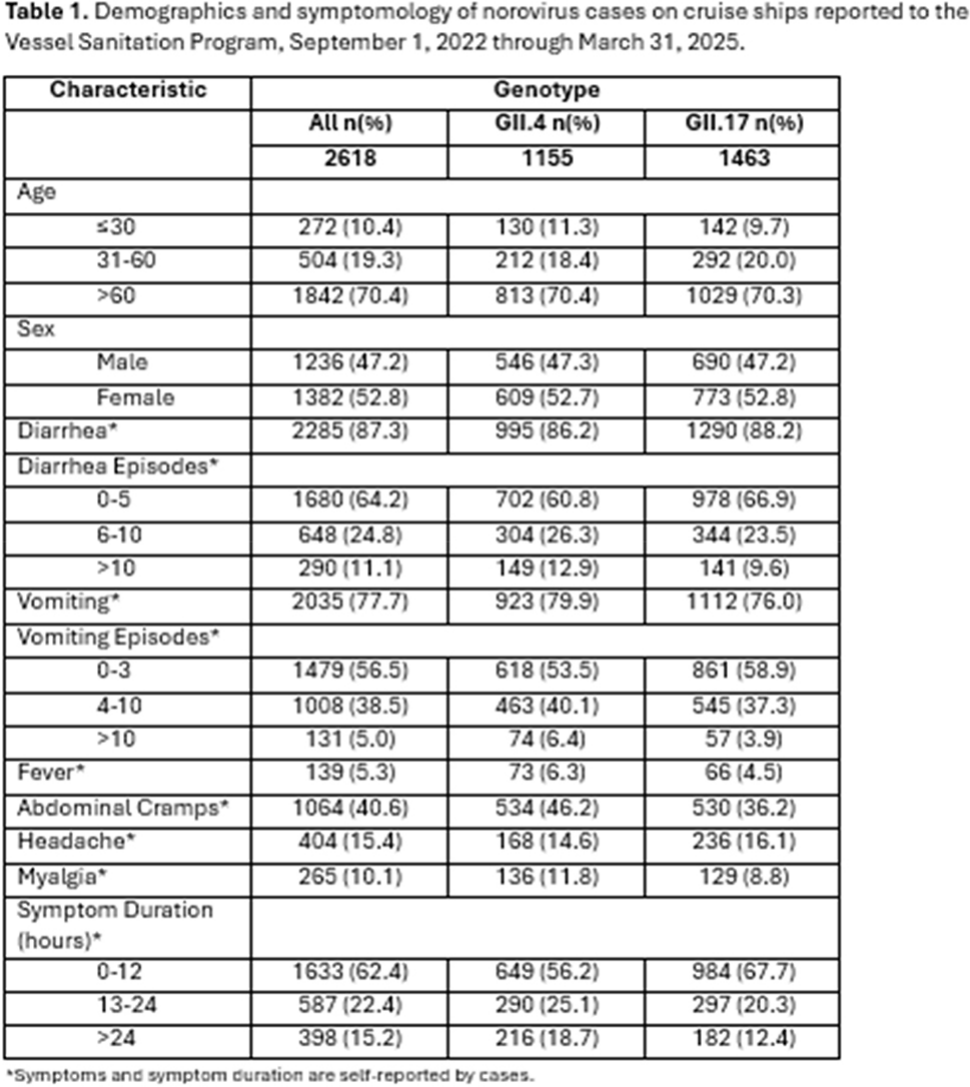
Demographics and symptomology of norovirus cases on cruise ships reported to the Vessel Sanitation Program, September 1, 2022 through March 31, 2025.

**Figure:**
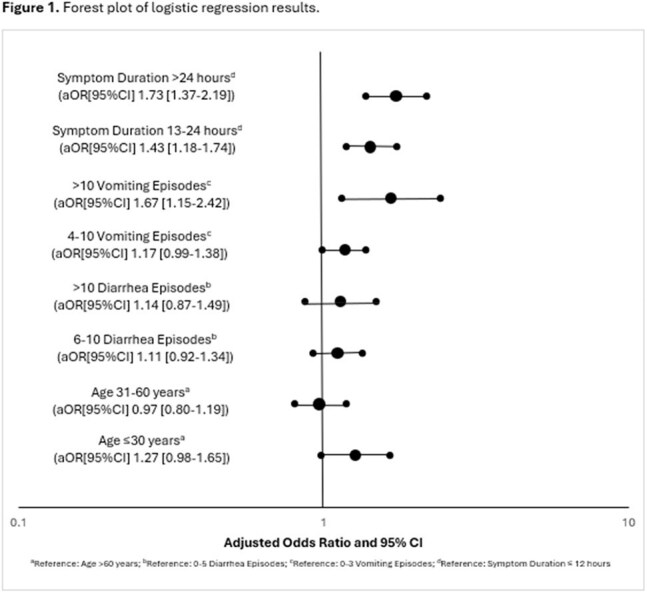
Forest plot of logistic regression results.

**Methods:**

We reviewed outbreak data sent to the VSP for the norovirus seasons from September 1, 2022-March 31, 2025 to identify outbreaks caused by GII.4 or GII.17. Outbreaks caused by > 2 norovirus genotypes concurrently were excluded. Information on symptoms, symptom onset and duration, and case demographics were collected. Descriptive statistics were calculated, and logistic regression was performed to compare disease characteristics and severity.

**Results:**

During the 2022-2025 norovirus seasons, we identified 2,618 cases for analysis. Most cases (n=1,842, 70.4%) were > 60 years old, and frequently reported the symptoms of diarrhea (n=2,285, 87.3%) and vomiting (n=2,035, 77.7%), with most cases reporting 0-5 episodes of diarrhea (n=1,680, 64.2%) and 0-3 episodes of vomiting (n=1,479, 56.5%). More than half of cases reported symptom resolution ≤ 12 hours (n=1,633, 62.4%) (Table 1).

Cases identified during GII.4 outbreaks were more likely to experience > 10 vomiting episodes (aOR: 1.67, 95%CI: 1.15-2.42) compared to GII.17 outbreaks. Symptom duration of 13-24 hours (aOR: 1.43, 95%CI: 1.18-1.74) and > 24 hours (aOR: 1.73, 95%CI: 1.37-2.19) was more common in cases identified during GII.4 outbreaks than those in GII.17 outbreaks (Figure 1).

**Conclusion:**

During the 2022-2025 norovirus seasons, cases in outbreaks associated with GII.4 had more vomiting episodes and experienced symptoms for a longer time than cases in outbreaks associated with GII.17. The findings of this analysis can inform patient management, staffing capacity, public health messaging, and infection prevention and mitigation measures during norovirus season.

**Disclosures:**

All Authors: No reported disclosures

